# Gendered ageism among women undergoing mastectomy across two life stages: early adulthood and later life in Pakistan

**DOI:** 10.1186/s12939-026-03082-9

**Published:** 2026-10-23

**Authors:** Tauheed Zahra

**Affiliations:** Institute of Sociology (Emmy Noether Group “Linking Ages”), Goethe University, Campus Westend, Frankfurt am Main, Germany

**Keywords:** Mastectomy, Gendered ageism, Self-perception, Phenomenology, Pakistan

## Abstract

This qualitative study examines how gender and age shape breast cancer and mastectomy experiences among women in Pakistan. Using critical phenomenology and drawing on 12 in-depth interviews with women across two distinct age groups (early adulthood and later life), the study identifies three key themes regarding age- and gender-based inequalities that shape women’s access to care. These inequalities are produced not only within the healthcare system but also through social and familial structures: [1] From Surveillance to Neglect: Age-Stratified Gatekeeping and Delayed Diagnosis, [2] Sacrificial femininity and internalized ageism, [3] Mastectomy as age-stratified embodied threat. Findings show that men act as financial and moral gatekeepers, limiting women’s healthcare access, especially for young married women regarded as reproductive assets. Older women experience internalized ageism, viewing their bodies as less worthy of care. Although participants reported no age-based discrimination from healthcare providers, their accounts highlighted that gendered ageism operates strongly in pre-clinical family and community contexts, determining who is allowed to seek care and when. The study demonstrates that the most critical barriers to timely breast cancer treatment are not biomedical but social, cultural, and age gendered.

**Supplementary Information:**

The online version contains supplementary material available at https://doi.org/10.1186/s12939-026-03082-9.

## Introduction

*I was sitting in the waiting area of a public cancer hospital in Pakistan known as the “healing space*,*” collecting data for my PhD thesis on the lived experiences of mastectomy patients. Suddenly*,* the silence became louder. A man came shouting at his wife*,* saying she had wasted his time*,* he had to go to work*,* and he had no time for her. The “healing space” instantly became a stage where a woman stood helpless*,* facing the possibility of a deadly disease. Despite her worry about her health*,* her face showed hidden pain as she stood motionless. In that moment*,* inequalities and power relations within the family became visible. Her silence and her husband’s reaction revealed the unequal power dynamics shaping women’s everyday lives and their access to healthcare*

This moment stayed with me throughout my research. The inequalities women faced reaching to the healthcare settings are not confined in clinical settings but before that, in the doorways, waiting rooms etc. This was one of the moments, I observed but it’s not always like that, some women accompany their husbands, sons, father who waited patiently, they do ask information on behalf of them. Others come alone, deprived, were rushed as this woman did, quietly absorbing the diagnosis and situation. Her silence was not simple, it explains a broader pattern which I would go on the trace throughout the study, which helps me to understand how age and gender together shape whose life is worth treating. Studies have been done to understand gender disparities and age-based discrimination in healthcare, but there are few studies which explore how these factors intersect to shape women’s experiences in low and middle-income countries like Pakistan. Women’s experiences during breast cancer and mastectomy remain underrepresented in the literature, this study will help to fill the gap by centering the lived experiences of Pakistani women having mastectomy during the breast cancer journey.

This paper will examine the lived experiences of women from two different age groups early adulthood and later life seeking breast cancer care. Drawing on the concept of gendered ageism, It helps to examine the intersection of age- and gender-based discrimination as an analytical lens to understand how women’s access to healthcare is systematically devalued across early adulthood and later life [1]. Pakistan offers a unique context where social structures and cultural barriers intersect with the healthcare system and strongly influence women’s experiences of breast cancer care.

Against this backdrop, the paper asks: How do age and gender intersect to shape women’s experience to breast cancer care and mastectomy in Pakistan, and how do these intersecting factors produce structural inequalities in access, treatment, and social recognition? Mastectomy is a surgical procedure in which all breast tissue is removed and is a treatment option available in breast cancer journey to prevent recurrence. To address this question, I proceed in three steps. First, I outline the current situation of breast cancer care in Pakistan. Second, I examine how social and cultural barriers shape women’s experiences and access to healthcare. Finally, I analyze how these factors intersect and contribute to structural inequalities within the healthcare system.

## Breast cancer care in Pakistan and structural inequalities

In the global scenario, more than one million women are diagnosed with breast cancer [2], making it the most commonly diagnosed cancer among women worldwide [3]. According to the latest GLOBOCAN 2022 report [4], female breast cancer is increasing among low- and middle-income countries like Pakistan. The incidence rate is rising more rapidly in Pakistan compared to high-income nations, with an alarming increase in the number of cases diagnosed at younger ages [3]. The percentage of breast cancer among the young age group (30–34 years) has also increased in 2020 and 2025 compared to 2015 in Pakistan [5]. The incidence of breast cancer is notably high, positioning the country as having one of the highest rates of this disease among Asian nations [6].

Every one woman in nine is diagnosed with breast cancer. Drawing on data provided by the Shaukat Khanum Memorial Hospital Research Centre, it is estimated that there are approximately 83,000 new cases of breast cancer annually in Pakistan, with more than forty thousand women dying from the disease each year. This indicates that women in Pakistan diagnosed with breast cancer have a higher mortality rate than those in high-income countries, pointing to limited access to healthcare and delayed diagnosis [7]. This alarming statistic underscores breast cancer as the foremost cause of cancer-related mortality among Pakistani women [8]. With a total population exceeding 241 million, Pakistan faces a substantial cancer burden, with an estimated 185,748 new cancer cases and more than 118,631 cancer-related deaths in 2022 [9]. According to GLOBOCAN 2022 report [4], Pakistan has the highest cancer burden relative to its population size and healthcare resources among South Asian nations.

Despite the rise in breast cancer among women across different age groups, there is no proper national cancer registry in Pakistan The absence of a cancer registry is not only a technical issue; it prevents the tracking of disease trends, treatment outcomes, and health equity indicators, thereby obscuring inequalities in cancer outcomes [10]. Cancer registries play a vital role in providing population-based data on cancer incidence, mortality, and treatment efficacy, enabling health authorities to monitor disease patterns. The major reasons behind the absence of a cancer registry in Pakistan include limited resources, insufficient funding, and inadequate infrastructure [9].These resource constraints clearly reflect a broader pattern of systemic negligence in South Asian cancer control, where lack of investment in cancer prevention and early detection is evident [11].

On top of these challenges for quantitative and epidemiological research, the lived experiences of women navigating breast cancer care and mastectomy particularly in relation to age, gender, and socio-economic status remain unexplored. Most qualitative studies on South Asian women have been conducted in high-income countries such as the UK, USA, and Canada, focusing more on screening uptake rather than the lived experiences of women during treatment [12]. This study employs a critical phenomenological framework [13, 14] to understand the lived experiences [15] of breast cancer patients undergoing mastectomy within broader societal structures of inequality in cancer care. A critical phenomenological approach offers a methodological and analytical lens to examine how external forces are experienced, embodied, and interpreted by individuals [14]. Beyond biomedical framing, it helps conceptualize mastectomy as a materially and socially situated experience [16], uncovering how social norms, stigma, and inequality affect both physical and psychological well-being. By combining lived experience (phenomenology) with critical theory [17] as a structural lens, this study explores women’s subjective experiences and analyze how these experiences are shaped by broader social structures. I argue that mastectomy is not merely a biomedical event but a socially constructed phenomenon, shaped by women lived realities and the systemic barriers they encounter.

## Mastectomy as a socially situated experience: intersection of age, gender and structural inequality

Mastectomy^1^ remains the predominant surgical procedure available for breast cancer patients among low- and middle-income countries. In the South Asian context, breast-conserving surgery is used in only 23–48% of cases, compared to 52–77% for mastectomy [18].While in Pakistan context breast-conserving surgery accounts for a minority of procedures, with modified radical mastectomy/mastectomy comprising up to 68–70% of surgical interventions [19].In contrast to high-income countries, where breast-conserving surgery is the standard care for early malignancies. Research has also found that only 1% of women in Pakistan undergo breast reconstruction after mastectomy, compared to global reconstruction rates of 5–50% [20]. Pakistan faces exceptional cultural and social factors limiting Breast conserving surgery (BCS) as well as immediate breast reconstruction (IBR), and there is a need for deeper understanding and exploration of these barriers, including through awareness programs to increase patient acceptance [7]. Breast-conserving surgery and immediate breast reconstruction were rarely discussed or pursued by the study participants due to certain structural barriers: presentation at advanced stages and the substantial financial costs associated with post-BCS radiation therapy, which left radical mastectomy as the only affordable option for low-income families. Therefore, mastectomy is not only a biomedical intervention, but a phenomenon deeply shaped by intersecting systems of gendered ageism and structural inequality. This intersection determines the embodied consequences women experience across their life course [11].

In a country like Pakistan, the major reason behind the increasing proportion of women undergoing mastectomy is late diagnosis, produced through multiple intersecting factors that vary based on women’s age and life stage [7]. Critically, the meaning associated with mastectomy and its embodied experience differs by age in Pakistan [21]. Breasts are considered sacred parts of the body, and breast-related issues are not typically discussed openly among women. However, the reasons for silence and the consequences of disclosure differ fundamentally by age. In the cultural context of Pakistan, men are regarded as financial and moral gatekeepers, and the decision to undergo mastectomy is rarely in women’s hands.These patriarchal structures are influenced differently across different age groups and shaped how women from different age group experienced the embodied consequences of mastectomy. Women in early-adulthood (reproductive age) have their own insecurities; early diagnosis can disrupt marriage prospects and increase fears of being considered “useless” or “less attractive” after mastectomy. Older women, however, consider their bodies less important ; they internalize ageism: the process by which women adopt the belief that their bodies are less worthy of attention, protection, or healthcare resources [22]. Gendered ageism in healthcare may contribute to older women’s reduced prioritization of bodily concerns, in part through processes of internalized age-related beliefs that can shape how symptoms are perceived and acted upon. Silence among older women is self-protective, driven by the belief that disclosure would burden their adult children financially.

Younger women experience mastectomy as stripping away their sexual and reproductive identity, while older women perceive it as reinforcing the devaluation of their aging bodies [23].These factors lead to social stigmatization and isolation among mastectomy patients, making it difficult for women in Pakistan to access care and support after the procedure. Pakistan’s patriarchal family systems (extended, joint, and nuclear) [24] further shape women lived experiences. These intersecting familial and social dynamics indicate that breast cancer is not only a biomedical issue but also shaped by broader social structures within society. Existing literature focuses on clinical settings, the causes of breast cancer, and survival statistics; however, there is a significant research gap in understanding the qualitative experiences of women undergoing mastectomy within Pakistan’s structurally vulnerable and patriarchal context. This study adopts a critical phenomenological approach to fill this gap.

## Theoretical framework: a phenomenological approach to gendered ageism in breast cancer care

The study draws on two primary concepts structural vulnerability [25] and gendered ageism [26] which are examined through a critical phenomenological lens to understand the embodied impact of intersecting social forces.

Structural vulnerability [25], a concept derived from critical public health literature, shifts the focus from individual behaviors to the systematic ways hierarchies of power are embedded in social, political, and economic structures, exposing certain populations to compounded health risks and worse health outcomes [27]. Structural vulnerability identifies which populations bear the heaviest burden of preventable harm and how systems of oppression actively constrain the range of choices available to them. In Judith Butler’s theorization of vulnerability and precarity, she describes vulnerability not as an individual failing but as a fundamental human condition distributed unevenly across populations through institutional and structural arrangements [28].

In the context of Pakistan, structural vulnerability operates through intersections of economic, patriarchal, and healthcare hierarchies that expose women to different health risks at different life stages. These structures include unequal access to healthcare, men control over women’s economic autonomy, the absence of cancer registries, and cultural norms that position women’s bodies as secondary to family obligations [29]. Public health literature illustrates that inequalities are not only embedded within institutional policies but are also deeply rooted in social structures, family relations, clinical encounters, and economic transactions. These dynamics determine which bodies receive care, which symptoms are taken seriously, and whose survival is considered worth protecting [27].

The second primary concept used in this study is gendered ageism. The term is widely traced to the work of Itzin and Phillipson 1995, who examined age related barriers mainly with in organizational settings and workplace marginalization to explain the double jeopardizing of age and gender among women [26, 30], while the concept has subsequently been extended into social psychology, body image and more recently into healthcare and health research [31], but the field remain small and largely descriptive, so the concept of gendered ageism is still under-theorized and empirically underdeveloped [30].

In this study, gendered ageism is used as an analytical extension rather than an established healthcare specific framework for understanding breast cancer and mastectomy care in Pakistan. It serves as a lens through which to examine how gender norms shape the meaning and consequences of age-based devaluation in women’s mastectomy journey. This approach also consider how ageism is experienced differently across genders and shapes women’s social, economic, and bodily experiences [32, 33].

The study’s accounts show that women at different life stages described different constraints and pressures. Younger women reported concerns regarding seeking healthcare without permission from husbands, fathers, brothers, reflecting the gendered expectations surrounding women’s autonomy. They also talked about marriageability, stigma and the implication of mastectomy for marriage prospects. Older women by contrast often receive less attention within the family, underestimate their symptoms, or internalize self-ageism [34] developing stereotypical and self-biased assumptions about themselves. They often consider their health and bodies “useless” or “less important.” At this point age related assumptions intersects with gender constraints, in certain situations women experience different forms of disadvantage that reflects the interaction of age and gender. However, the study findings are specific only to the interviewed women and should not be interpreted as an evidence against all younger or older women experiences these pressures in same way.

In the south Asian healthcare context, a huge data set is available which talks about the generic structural vulnerability but alone this cannot explain why it’s different across different life stages. Gendered ageism therefore adds that age differentiated dimensions by showing how economic, patriarchal, and age-based hierarchies operate together and shapes women’s access towards treatment and social recognition. This framework, therefore, helps to analyze how structural inequalities such as age and gender creates pre-clinical barriers to breast cancer women undergoing mastectomy among two different age groups in Pakistan? and how do these intersecting factors produce structural inequalities in access, treatment, and social recognition? It also helps identify how gendered ageism creates specific risks for women experiencing breast cancer, and how socioeconomic inequalities compound these risks, resulting in limited access to cancer care.

## Methodology: a qualitative study of mastectomy patients in Pakistan

The research that this paper draws on is based on qualitative methodology. Data collection was conducted in four public cancer hospitals located in two metropolitan cities, Islamabad (the capital) and Lahore, the largest population center in Punjab. These cities were selected because they are among the most frequently visited locations for cancer care, exhibit socio-economic and regional diversity, and report a high ratio of cancer patients.

### Positionality and reflexivity

As a Pakistani female researcher pursuing my research at a European institution, my positionality uniquely shaped the fieldwork dynamics and data collection. I faced multiple challenges while reaching out to study participants; it took more than six months to clear organizational matters, including security clearance and IRB approval from the hospitals. Being a native speaker, as well as a woman in the field, allowed me to communicate easily with respondents, fostering an immediate sense of shared identity, cultural understanding, and trust. They considered me a professional whom they were unlikely to see again outside the context of the research, which may explain why they were more open with me than with their own family. My appearance and manner were essential in breaking the ice, and this rapport helped overcome traditional barriers of shame and silence around female bodily anatomy and breast cancer. However, my affiliation with a foreign university sometimes positioned me as an academic outsider, which encouraged participants to explain cultural norms such as familial restrictions and gatekeeping structures explicitly, rather than assuming they would be taken for granted. Throughout the data analysis, I remained highly reflexive, consistently evaluating my dual outsider/insider status to avoid imposing external biases on participants lived realities.

### Data collection procedures

In-depth interviews were conducted using a semi-structured interview guide and extensive field notes. The full interview guide, developed as part of the author’s broader PhD project exploring the lived experiences of mastectomy patients across the life course, is provided as a supplementary file. The interview guide was structured to facilitate participants in narrating the complete illness journey. Each interview lasted 60–90 min. All interviews were conducted by the author, a native Urdu/Punjabi speaker, which allowed participants to speak in their first language. To ensure participants’ data safety and confidentiality, interviews were conducted separately from family members; in cases where caretakers were present, they were offered separate time upon request.

### Data translation and analysis

Interviews were transcribed manually in Urdu and later translated into English by the author. While translating from Urdu to English, a back-translation check was carried out to confirm that the translation preserved the accurate meaning and idiomatic nuance of participants’ original wording. To ensure deep interpretive immersion and close engagement with the data, interviews were transcribed manually without the use of any qualitative data analysis software, following the six-step coding guide by Braun and Clarke [35].

### Participant sampling

For participant recruitment, a purposive sampling technique was used (*n* = 12) on women at two distinct life stages who had gone through mastectomy in breast cancer. The purpose of this sampling technique was to understand the diverse lived experiences of women from different socio-economic backgrounds, their access to healthcare facilities, and the common issues and challenges faced throughout their breast cancer care. This manuscript draws on the subset from the author’s broader doctoral project, exploring the lived experiences of mastectomy patients across two life stages (early adulthood and later life). The participants included in this study’s analysis are those who had undergone mastectomy, and this manuscript focuses on the embodied and social experiences following this procedure, rather than on comparative surgical procedures such as breast-conserving surgery or immediate breast reconstruction.

Age was one of several intersectional categories alongside gender and socioeconomic status considered from the outset of the broader PhD research. However, as coding progressed, age emerged as a particularly salient axis of differentiation, and this manuscript focuses specifically on that dimension. To capture a sharp empirical contrast in social positioning, financial autonomy and family roles, the sample strictly targeted two distinct age cohorts (as detailed in five-year age bands in Table 1): early adulthood (ages 20–40, * n=5*) and later life (ages 65–70, * n=7*).

Women in early adulthood are often situated in phases of career formation, active childbearing, and marital negotiation, where the loss of breast carries stigma regarding body imaging and marriageability. However, older women more commonly navigate post-reproductive roles and increased health-related vulnerabilities and the deprioritizing of their health within the household. The inclusion of these age groups ensured that the study captured the first-hand experiences of women navigating the healthcare system at these distinct ages.

The selection of these two age groups was based on the theoretical differentiation and the hospital registration data provided by the hospitals prior to the participant recruitment at the time of the study. Based on the available data at the time, these two age groups were identified as particularly vulnerable and underserved, capturing the lived experiences of women having mastectomy during their breast cancer journey. Although, epidemiological data show that in Pakistan women around aged 41–64 represents the largest population affected by breast cancer [5], this study utilized theoretically driven purposive sampling to examine distinct life-stage extremes. The exclusion of the middle-aged cohort is a recognized structural bound of this dataset, as detailed in the study limitations.

### Ethical considerations and anonymity

To follow the institutional review guidelines Table 1 explains the age ranges rather than exact values to protect the participants anonymity in such a small qualitative sample. Exact numerical ages were converted into the five-year bands presented in Table 1. All respondents were provided informed consent in both (English/Urdu) and an information sheet outlining all study details, including the consent of using data anonymizing in research publications. To ensure anonymity, participants were not asked their original names, the demographic table presents ages in brackets. I paid particular attention towards participant emotional well-being and questions were asked after proper ice breaking and providing space to participants did not feel pressure or judged.

The study approved by the relevant ethical committee Ethics Review Board of the Department of Social Sciences, Goethe University Frankfurt (Germany).

### Sample description

A socio-demographic characterization of the participants is presented in Table 1. Patients were recruited from various socio-economic backgrounds, classes, ages, marital status, family settings, and educational levels. Only generalized demographic information is presented to protect participant anonymity.

**Table 1:** Socio-demographic characterization of participants (*n* = 12)

Participant ID	Age Range	Marital Status	Education	Family Type
P1	25–30	Single	Postgraduate education	Nuclear^2^
P2	20–25	Married	Undergraduate education	Joint^3^
P3	35–40	Married	Postgraduate education	Nuclear
P4	30–35	Married	Secondary education	Nuclear
P5	65–70	Married	Primary education	Nuclear
P6	65–70	Married	Postgraduate education	Nuclear
P7	65–70	Widow	Primary education	Nuclear
P8	35–40	Married	Primary education	Joint
P9	65–70	Married	No formal education	Nuclear
P10	65–70	Widow	No formal education	Nuclear
P11	65–70	Widow	No formal education	Joint
P12	65–70	Married	Secondary education	Nuclear

^2^Nuclear families in Pakistani household are those married couples with the children living separately from their extended kin

^3^In Pakistan, a joint family refers to a co-residential household comprising married sons, their spouses, children, and parents living together under one roof

### Data analysis

Reflexive Thematic analysis [36] was applied for analyzing the data collected through in-depth interviews. It is a method which helps the researcher to identify, analyze and interpret the patterns in data, their meanings which refers as themes within the qualitative data [37]. Therefore, guidelines for reflexive thematic analysis were employed which included familiarization with the data, generation of codes, development of initial themes from the codes, review and editing the initial themes, naming and defining of themes, familiarization with the themes and production of reports [35]. Data analysis begins by identifying the initial coding emerged by the reading of interview transcripts, followed by that were notable and reflected in literature. Deductive and inductive codes were generated by analyzing the common response patterns into the major overarching themes [38]. Second, cycle coding helps to identify the conceptual and thematic similarities among codes which results in overarching themes. Lastly the identified themes were named after multiple revisions and compared with the existing literature on the lived experiences of mastectomy patients during breast cancer journey.

The study identified three interrelated key themes:

1. From Surveillance to Neglect: Age-Stratified Gatekeeping and Delayed Diagnosis.
2. Sacrificial Femininity and Internalized Ageism.
3. Mastectomy as an Age-Stratified Embodied Threat.

## Findings

### Theme 1: From surveillance to neglect: age-stratified gatekeeping and delayed diagnosis

Under this theme, I discuss the mechanisms influencing delayed breast cancer care among women from both age cohorts in Pakistan. Multiple structural factors contribute to delays in diagnosis, however, following section describes how age and gender play role in women access to health care and how it shapes their experiences. The intersection of structural vulnerability and surveillance within the household structures.

Throughout the sample among mostly younger married cohort (age 20–40) the requirement for male authorization for healthcare access emerged as foundational base line reality. Participants consistently required permission prior to seeking medical evaluation. This required permission dictates when and how they respond towards the early symptoms, as illustrated in the following account.

*I didn’t go outside without my husband’s permission. The day I felt there was something in my breast*,* my husband was not home—he went for some Islamic gathering in Lahore (another city) and I didn’t want to make him worried. I thought I was having my periods*,* that’s why… Without his permission I can’t step out from home. He came back after a month… then I told him about the cyst. He said*,* follow what my mother said… (Age*,*35–40)*

In this account the participant interpreted the lump as a normal part of her “period” and delayed disclosure until her husband’s return. For younger married woman, her health conditioned and her body was socially positioned in the category of wifehood, where marital and generational obedience and reproductive responsibilities shape the healthcare decisions. When she disclosed it to Husband, he transferred the situation to his mother, where her survival requires multiple layers of approval for generational authorization. The late disclosure, passing through other family members, all these layers processes are described by several participants in this age group.

*I got confused because I was only 39 years old and were unsure whether I needed a mammogram or not… (Age*,*35–40)*

*I kept thinking that I am so fit*,* so healthy and young—there are no underlying issues… So how can this happen? I thought it’s a breast injury because I fell on this side (Age*,*35–40)*

These accounts demonstrate that how younger age group thinks that it’s too young to have breast cancer and leading them to deny the medical tools and requirements to go further to diagnosis. Most participants struggle to recognize symptoms in early stages due to the age-related preexisted assumptions. They are in clinical denial phase to accept the possibility of having cancer or something serious at this stage because they associate youth with reproductive functioning, physical strength and inherit health. Rather than considering the symptoms serious, they explained through alternative socially acceptable meanings for example one participant assumed lactation prevent from cancer, another explained might be injury because I fell on the floor. Together from these narratives it illustrates that women and healthcare encounters both can be seen through the younger age lens to interpret breast related symptoms.

Similar experiences emerged from other interview partners from the younger age group (married), who described that they need male approval for even “stepping out from the home” or any clinical help. These women accounts extract that medical delays were not their personal choice but enforced by the cultural requirements of marital obedience, where the agency of seeking help is not in their hand not because the social structure reinforces but because of their younger age group. The empirical findings from mastectomy patients clearly define day-to-day structural inequalities produced by patriarchal control.

*I recently got married; my husband lives in Spain. When I told him about the cyst in my breast*,* he asked me not to share it with anyone and did not allow me to go to the hospital for a checkup… He says that he doesn’t want anybody to see his wife… I do ‘sharia pardah’… nobody can even see my eyes. I used to be covered properly from head to toe… I waited for seven whole months and when the pain was unbearable*,* I told my mother and went to the hospital with her… later I was diagnosed with breast cancer. (Age*,*25–30)*

This participant’s account describes the control of her husband from outside the country on her visibility and mobility, including his refusal to seek healthcare which directly contributes to delay her access to care for seven months later sought through by her mother. This situation reflects the pattern describes by the several younger married women, where husbands control their decisions to disclosure and seeking healthcare.

*I went to the local healthcare center where the doctor said that it’s a duct gland because you are lactating. After some time*,* I couldn’t move my left arm*,* so I went to the big hospital in the city and was later diagnosed with cancer… (Age *,*25–30)*

In contrast, most older women illustrate different patterns. This set of data didn’t experience delays because of restriction from others, most of them describe the normalization and minimization of the symptoms as an expected part of aging as shown below.

*I felt a lump in my breast. I don’t have my husband—I am a widow. I told my neighbor; she is an old lady. She said the pain must be gastric and told me not to go to the doctor*,* that they just make money… So I went to the herbal practitioner near my house. He gave me tablets for six months without checking me… later I had severe pain in my arm*,* I couldn’t even hold a glass of water… By chance I went to the doctor; she referred me to the public cancer hospital*,* where it was diagnosed that I have breast cancer. (Age *,*65–70)*

*I am an old lady already…I know getting old will make the body parts weak…its just because of getting old… (Age *,*65–70)*

*I am moving on my own…its enough for me at this age.i am going to washroom on my own.i pray*,* recite Quran on water later drink it…(Age *,*65–70)*

Older women experiences illustrate that how age and gender play role to shape the response towards breast cancer symptoms. Here, the state’s role in the life of a widow demonstrates structural neglect produced through failed navigational systems rather than gatekeepers alone. These hindrances are pre-clinical barriers, occurring before women access any formal healthcare. In contrast to younger women, older participants did not neglect or dismiss the symptoms because of being too old and they normalize those changes as a part of ageing bodies. These accounts illustrate the intersection of gender and age in later life. Women in their post reproductive bodies are considered as less socially valuable, symptoms at certain age got less attention by themselves as well as from the people around them, rather than requiring urgent attention symptoms are considered as ordinary.

However, these findings suggest that women in both age groups represented in this study face multiple barriers both inside and outside the household. Lack of healthcare navigation and men acting as gatekeepers significantly delay diagnosis. However, age comes into play not only as it determines women’s autonomy but also because it shapes an entirely different type of exclusion. While younger women delayed diagnosis due to active male surveillance, other hand older women accounts describe the inclination towards more on the informal advice from the family members, neighbors or herbal practitioners rather than the structural referrals, by which diagnosis delays for several months. By Centering the gendered ageism concept, empirical findings of the study illustrate that in Pakistan patriarchal oppression is age certified, shifting from overly controlled in younger age to social neglect and post reproductive bodies irrelevance in later life.

### Theme 2: Sacrificial femininity and internalized ageism

Under this theme, I discuss how women across different age groups prioritize their family needs over their health and how they internalize the idea that their health is negotiable and less important than that of their husbands or children. In countries like Pakistan, cultural norms of female sacrifice and compromise are widely documented [39] And Women’s health is often considered secondary to household and family needs. Most Participants from both age groups (early-adulthood and older age) considered their bodies and needs “less important “from other family members. Furthermore, my material indicates that across class and education, women share similar thought patterns. Throughout the interviews women claims to fulfill the family duties first as one of the patients delayed her chemotherapy because she has to prepare for her daughter’s engagement, stating that she would be “worthless “if she could not fulfill the mother’s role.

*How could I tell my husband to spend all that money on me? My daughter was studying. We don’t have a lot of money*,* but both my son and my daughter are studying very well. I didn’t talk about my pain to my husband because we have to pay their fee… I don’t want to burden my husband… A mother’s duty is to sacrifice. My health can wait. (Age *,*35–40)*

This participants narrative describes the disclosure of her pain from her husband to protect her children’s school fee and don’t want to burden her husband, framing this situation as a mother’s duty. Several other participants describe similar situations and hide their symptoms, not putting any emotional and financial burden on their families. The findings act as a critical bridge to explain how women sacrifice themselves during illness.

How does age matter here? Women in the younger age group bore the burden of maintaining the infrastructure of care for future generations, considering their own bodies secondary to their families. In contrast, older women view their bodies through the lens of internalized ageism, which shapes how they interpret bodily changes and seek care during breast cancer. This age group is mostly financially dependent on their male children, and they internalize this dependency, feeling they are a burden. Breasts are considered sacred and private parts of the body, and older women impose neglect upon themselves due to traditional mindsets. Moreover, discussing breast-related issues with family was viewed as shameful by many of the women I talked to. This cultural barrier intersects with age and reinforces silence.

*I have only one son*,* and I lost my husband a year ago… He has four children to feed. My cyst is not new; it’s almost two years old*,* but I didn’t tell anyone. I just prayed it would go away on its own. I don’t want to create any difficulty for my son… I have lived my life… Cancer is very expensive… I got initial treatment on the Sehat Card*,* but now the government stopped it; I don’t know for what reason… We don’t have money for further treatment. My son has savings for his children’s future. My life is not worth that sacrifice… I am around 70 years old… I just asked him to take me home. I don’t need any treatment. (Age*,* 65–70)*

In this participant case she declined further treatment, by weighing her treatment against her son’s financial future. Similarly, many of the accounts from this age group didn’t give value to their own health and preferred financial preference of their children. Her statement of “My life is not worth sacrifice” demonstrates that how internalized ageism operates in practice. In this context what I termed as internalized sense of having “disposable” where participant perceived themselves as less important, and less worthy of attention is not an abstract label, but a lived reality reported by older participants. Gendered ageism shapes these situations, younger women’s symptoms are controlled and silenced; some older women may internalize the belief that their bodies are not deserving of care, leading to delayed or refused treatment.

### Theme 3: Mastectomy as age-stratified embodied stigma

Mastectomy in the breast cancer journey is not just a medical event, but an embodied experience of gendered ageism, where women’s bodies are valued or devalued based on reproductive expectations [40]. The meaning of bodily changes that occur during breast cancer contain different meanings across the life course: younger women see mastectomy as a threat to their femineity, sexuality and reproductive value while older women experience it as less valuable within the social context where the aging bodies are seen as post reproductive.

By embodied experience, i refer to how women feel and interpret the loss of their breast during the breast cancer journey across different age groups. Younger women see it as a disruption to their femininity, beauty, and reproductive identity, while older women may experience their bodies as post-reproductive and less worthy of attention and care within the social context where ageing bodies are considered less valuable. However, in both cases, gendered ageism is felt through the body itself, including the scars, the absence, and the social responses toward these changes.

The procedure runs the risk of disrupting women’s self-understanding of identity, agency, and continuity within contexts of gender and age [41]. Mastectomy can contribute to negative body image, as breasts are symbols of motherhood, sexuality, and femininity. It affects socially rooted expectations of women’s bodies across different ages [42]. Losing one or both breasts produces what researchers describe as “liminality”—a state of being caught between normal life and post-diagnosis life—leading to ambiguity about self-identity and the meaning of living without breasts [40]. The liminal state comes when women do not consider themselves in their previous social roles, as well as not in their pre-cancer bodies. They occupy an in-between condition, not recognized as healthy and whole in their new embodied body.

These experiences are not only subjective but reflect disparities in healthcare systems and patriarchal structures that systematically fail to provide women with safety, dignity, and bodily autonomy [43]. Stigma associated with mastectomy constitutes a form of cultural violence embedded in social structures that judge women based on appearance and reproductive capacity [44].

The women aged 20–40 undergoing mastectomy in my sample experience it as an embodied threat that jeopardizes social survival. Their bodies are positioned as reproductive commodities in the marriage market. This commodification is described by scholars as “gender socialization,” encouraging women to see their bodies as objects evaluated by men and tied to reproduction [42]. Bodily loss becomes stressful when it disrupts marriage prospects. Two prominent cases of unmarried women aged 23 and 26 revealed that they hid their disease from everyone. One was engaged, and her fiancé and in-laws had no idea about her condition. Their families believed that a diagnosis made them “useless” and “damaged” in the marriage market.

One participant said:

*I lost my hair*,* my eyebrows… I didn’t come out of that trauma*,* and the doctor asked me to go for mastectomy because otherwise they cannot remove the cancer… I wasn’t ready for this. I just wanted this test from Allah to end… Now I lost one breast… I felt that I lost my life… I am living in my home like a thief… Nobody even shakes hands with me except my immediate family… My mother always hides me while going to the hospital. She has a lot of fears that nobody will marry me now… I am not tired from cancer but from this fear of people. It is unbearable…( Age 25–30)*

Another participant said:

*I was engaged when I got diagnosed with breast cancer*,* and I had mastectomy done… My in-laws know about my cancer but not about my mastectomy… I always wanted to discuss it with my fiancé*,* but my family never allowed me. They said*,* “We don’t want any disaster in your life*,* we want you to marry and later he will know on his own… I am ready for the consequences. I know he will leave me whenever he knows about my mastectomy.” … (Age *,*20–25)*

Both findings illustrate how social structures assign high social and economic value to young women as prospective wives and mothers. One of the participants, concealing her mastectomy from his fiancé, fearing it would end the engagement. Another, the unmarried one conceals her symptoms more broadly, fearing it would affect her prospects of marriage. Mastectomy is seen as stripping the body of its value as a social commodity. Women’s bodies are treated not as their own but as social and economic assets [45]. These experiences show that beyond medical implications, mastectomy becomes an embodied disruption threatening women’s sense of self and social legitimacy.

Previous research has reported that women of both age groups face gendered discrimination and systemic barriers, but the nature of the embodied threat differs. Older women are more likely to be diagnosed with advanced cancer, receive less aggressive treatment, and have poorer outcomes [46]. Older women in this study often have less knowledge about breast cancer care, described as shame associated with breast related discussion and face multiple barriers; they may not recognize early symptoms or initiate healthy behaviors because they believe that at their age, its normal or treatment is no longer worthwhile. They normalize breast cancer as a part of aging. Women internalize self-ageism and consider their bodies less important.

One participant explains:

*I feel ashamed to talk about it to my sons. I am a widow and I only have three sons. You know talking about the breast to the son is very difficult; I felt that may be its normal because I am ageing… and I live in a village; there is no doctor near my home…so I just accept it as the part of my aging (Age *,*65–70)*

This account described how internalizing self-ageism, geographical accounts and shame associated with breast related discussion led her to interpret the symptoms as the common symptoms of aging. This is not individual acceptance of aging but reflects culturally embedded ageist messages in healthcare systems that devalue older women’s survival [11].This internalization reinforces that the older bodies deserve less attention and adopts silence over health needs, creating powerful pre-clinical barriers that prevent older women from seeking timely breast cancer care.

The missing narratives related to breast-conserving surgery and immediate breast reconstruction do not indicate that participants rejected these options; rather, most participants in this study reached clinical care at advanced stages and were treated at tertiary care hospitals, where mastectomy was recommended based on their disease stage. This left limited opportunity within this dataset to discuss alternative approaches.

## Discussion

This critical phenomenological study questions the biomedical framing of breast cancer care as purely biomedical by indicating that delayed diagnosis and treatment are deeply rooted in overlapping structural inequalities of age and gender. Grounded in the literature on delayed presentation in Pakistan [47], findings identify two analytically distinct, although interconnected barriers. General healthcare barriers include family restrictions on seeking healthcare, rural access problems, financial reliance, and purdah. While on other hand breast and mastectomy-specific barriers, which include constraints regarding male authorization for surgery, hesitation or cultural taboos around consulting male physicians or exposing the body, body image loss, limited treatment information and marriage-market / marital rejection stigma. Some of the barriers may overlap such as family restriction to consult male doctor in both categories. However, study findings align with the theoretical grounding of gendered ageism and help illustrate that age and gender do not operate independently in healthcare settings. Rather, age shapes the intensity and form of discrimination women experience [48]. In this study, younger women experience patriarchal surveillance and require approval due to their age to seek medical care, while older women face systematic neglect that reflects the devaluation of post-reproductive bodies. Patriarchy as a concept alone would explain why men act as gatekeepers and control women’s access to care in this study, but it does not cover the aspect of why that control took different forms across the two age groups for example, hyper surveillance for the younger age group and neglect in later life. Similarly, structural vulnerability explains the barriers women face in this environment, but it does not explain why younger and older age groups respond to these risks in completely different ways. Gendered ageism provides a framework to understand how this discrimination shifts based on women socially assigned reproductive value at each life stage. In this study the younger women minimized or denied the cancer risk by invoking markers of youth and reproductive vitality. For instance, they explained that breast feeding, physical activities, and general fitness excluded them from the possibility of serious illnesses like cancer. In contrast, the older women normalize the same symptoms as the part of their aging bodies. Gendered ageism is predominantly studies on older population, through these findings it illustrates that it is a life course phenomenon, where age functions as an interpretive lens, through which women from any life stage make sense of bodily changes. While age-based delays in diagnosis among younger women have been documented empirically, this pattern is rarely framed through the lens of gendered ageism, a concept traditionally developed around the older population. This study contributes by extending the concept of gendered ageism across two distinct life stages (early adulthood and later life). This resonated very well with the linking ages framework [49] which define age as not a fix category but as something socially and materially constituted through the life course, by comparing how age boundaries are drawn at different life stages. Reading through this lens, study findings illustrate that un/doing age within the context of breast cancer takes the forms of surveillance in younger age and neglect in later life, different practices doing the same underlying work.

These empirical findings support and extend previous research indicating that delayed diagnosis is not solely due to limited access to healthcare but also reflects social hierarchies, gendered control of household resources, and age-based devaluation of women [7]. Gendered ageism, therefore, operates as a pre-clinical hindrance, determining who is allowed to seek care and when. The second major finding confirms the influence of pervasive cultural norms [39]. Delayed care is not always imposed by patriarchy alone but also emerges from internalized systems in which women sacrifice their own health for their family’s emotional and economic security. This finding extends research on sacrificial femininity by demonstrating that sacrifice is not only cultural but also enforced by social institutions: participants experiences of navigating appropriate healthcare system, familial structures that position women’s health as secondary, and an economic system that denies women financial autonomy [50]. During the breast cancer journey, these broader sociocultural barriers related to healthcare intersect with the concerns specific to breast cancer and mastectomy, including body image, fear and stigma. Research conducted in Pakistan has similarly documented sociocultural, financial and healthcare related barriers contributing to delayed breast cancer presentation [47]. Older financially dependent women, such as participant (Age 65–70), argue that their lives are “not worth the sacrifice” and that their children’s savings should not be spent on them. Their narratives shows that self-neglect is not individual acceptance of aging but a consequence of institutionalized ageist devaluation [51]. Qualitative data regarding older women normalizing symptoms and deprioritizing their health align with broader epidemiological patterns in Pakistan. Hospital-based data, such as studies from Karachi, indicate that age-specific breast cancer rates peak between ages 50–64 [5]. Due to these socio-cultural dynamics, women in older age group are often under-represented and under-ascertained in national health registries, despite epidemiological data indicates that the women in this certain age group have a high risk of having breast cancer as many elderly patients fail to access or navigate modern healthcare systems. Consequently, internalized ageism and structural barriers prevent many older women reaching out to hospitals leads to late-stage representation or completely disappeared from the healthcare data [5, 52].

The study’s third contribution illustrates that mastectomy is considered an embodied threat particularly for younger, unmarried women, whose bodies are treated as reproductive commodities. In contrast, older participants described experiences of late diagnosis, and reduced treatment intensity, reflecting compounded age- and gender-based inequalities. This finding adds to mastectomy literature by showing how healthcare systems, social structures, and age-based devaluation influence whether women’s bodies are considered worthy of protection [11].

The study challenges existing explanations of cancer-related secrecy in Pakistan. Participants narratives suggest that silence functions as a survival strategy, where disclosure threatens marriage prospects, family stability, and economic security [21]. Women prefer silence not because they lack knowledge, but to protect themselves within a patriarchal structure where their value depends on remaining marriageable or maintaining family loyalty [53]. Finally, the findings suggest that women are not a homogenous group experiencing uniform discrimination, but that intersectional inequalities beyond the intersection of age and gender play a role. A young married woman facing patriarchal surveillance experiences a different form of neglect than a widow without financial autonomy. An illiterate woman in a rural area faces different structural barriers compared to an educated woman in a nuclear family. The empirical findings demonstrate that structural vulnerability emerges not from a single factor, but from the intersection of these positions [50].

This study fills a significant research gap in health literature. Existing studies on South Asian women’s breast cancer experiences have been conducted primarily in high-income countries (UK, USA, Canada) and focus mainly on screening uptake rather than treatment experiences. This study highlights the existing inequalities in health care structure including the absence of cancer registry and the dis-continuation of Sehat card reveals the structural inequities and gendered ageism operating within Pakistan’s healthcare system among women undergoing mastectomy for breast cancer. Ultimately, these findings demonstrate how pre-clinical barriers embedded in familial and economic structures shaped how and when the participants reached the clinical care during their breast cancer journey.

## Limitations

There are several limitations to this study. First, while the sample of 12 participants provides rich qualitative insights, it limits generalizability to the broader population of mastectomy patients in Pakistan. Second, because the study focuses exclusively on women who underwent mastectomy at four public cancer hospitals, it excludes: (1) women who do not seek biomedical care due to socio-cultural and financial constraints; (2) patients treated with breast-conserving surgery (BCS); and (3) women presenting with metastasized or advanced-stage disease for whom primary surgical resection is contraindicated. Third, the study focused on two distinct cohorts: early adulthood (ages 20–40) and later life (ages 65–70). The omission of mid-life cohort women ages (41–64) a group representing a substantial proportion of breast cancer cases in Pakistan (5) limits the extent to which the findings can capture experiences across the adult life course. Finally, geographic sampling was restricted to public hospitals in Islamabad and Lahore, potentially omitting the distinct experiences of women in other provinces or rural areas. Future research should incorporate the mid-life cohort, examine non-surgical treatment pathways, and include healthcare providers to better capture systemic inequalities within the healthcare system.

## Conclusion and contribution

This study explored how age, gender, and socio-economic status intersect to shape the experiences of women undergoing mastectomy for breast cancer at four public hospitals in Pakistan in two distinct age groups (early adulthood and later life), and how these intersecting factors produce structural inequities in access, treatment, and social recognition. Participants accounts highlight that age-based discrimination was not observed within clinical settings; women were treated similarly regardless of age once inside the system. However, gendered ageism was primarily located outside the clinic, rooted in familial, social, and cultural structures that determine who is allowed to reach the hospital.

Gendered ageism has mostly been studied among older age groups in Global North context [30], with fewer studies available on younger women. This study contributes to existing knowledge by moving beyond the concept of simple symptom delay: the exclusion is not within the clinical encounter itself, but begins earlier within the family, the hospital corridors, waiting areas, community and the broader social structures that shapes women pathways to healthcare. It provides an empirical map of how the female body is socially and economically valued in Pakistan: women have limited access to financial autonomy, men act as economic gatekeepers, and gendered ageism operates as a pre-clinical mechanism that restricts women’s access to care long before they encounter a physician. As a result, many women seek or receive medical attention only once their illness has already progressed to an advanced and financially devastating stage. The findings underscore that the most significant barrier to breast cancer care is not the disease itself, but a structural inequality in which women’s own health was not weighed as important and repeatedly deprioritized against their families financial and social needs rendering a woman’s body, in one participant’s words, “not worth the sacrifice.”

This study contributes to literature in two ways. First, by showing that gendered ageism is not experienced by older women alone or restricted to late life gerontological contexts, but may be operate across distinct life stages, including early adulthood and later life. Second, the study extends the application of gendered ageism framework within the healthcare context of breast cancer and mastectomy care, showing that it is not confined to clinical settings but may operate at macro and meso level throughout the journey of the disease.

## Supplementary Information

Below is the link to the electronic supplementary material.

Supplementary Material 1

## Data Availability

The datasets generated and analyzed during this study are not publicly available due to the sensitivity of the data and participant privacy. Data can only be shared in anonymized form upon reasonable request to the corresponding author (T.Z.).

## Footnotes

1 A mastectomy is a surgical procedure involving the partial or complete removal of one or both breasts, most commonly performed as a treatment for breast cancer.

## Declarations

### Use of AI Tools

During the preparation of this manuscript, Claude AI (Anthropic, San Francisco, USA) was used solely to check sentence structure and punctuation. The tool assisted in refining linguistic clarity and coherence without affecting the intellectual content, interpretation, or findings of the study. All the manuscript ideas, content and conception are my own and author takes full responsibility.
