# Supplementary material for: Gendered ageism among women undergoing mastectomy across two life stages: early adulthood and later life in Pakistan
Source: Int J Equity Health. 2026 Oct 23;25:254. doi: 10.1186/s12939-026-03082-9 (PMC13693215; doi:10.1186/s12939-026-03082-9)
Supplement: Supplementary file 1 [file 12939_2026_3082_MOESM1_ESM.pdf]

GOETHE UNIVERSITÄT FRANKFURT AM MAIN

LINKING AGES

VolkswagenStiftung

Section: Patient Information

**Gender:**	
**Age:**	
**Nationality:**	
**Occupation:**	
**Source of Income:**	
**Education:**	
**Marital Status:**	
**Number of Children:**	
**Type of Family:**	
**Date of Diagnosis:**	
**Treatment Duration:**	
**Years with the Disease:**	
**Rural/Urban setting:**	
**Hospital:**<br>**(Public/Private)**	

GOETHE UNIVERSITÄT FRANKFURT AM MAIN

LINKING AGES

VolkswagenStiftung

Interview Questions

I would like to know more about your journey / pathway with this illness. I am interested in everything you would like to share. So, when did all this start?

1.When and how did you first find out about this illness or any bodily changes?

- How old were you at that time?

2. Who was the first person you informed after finding out about this illness?
   - Do you remember the reaction and advice of the person you told first?
3. Do you remember which doctor, hospital, or traditional/spiritual healer you visited after finding out about this illness?
   - Who accompanied you?
   - How did you feel after that?
4. Which types of treatments were you informed about in your process of seeking medical help?
5. Do you remember when you were first informed about the option of mastectomy? How was that?
   - Who informed you about mastectomy?
   - What were your first thoughts when you heard this?
   - Do you feel like you were given sufficient information about mastectomy to make an informed decision?
   - What did others in your social circle (family, friends) think about this treatment?
6. How did you make the decision to go for a mastectomy?
   - Did you seek anyone's advice or permission for this treatment?

- Did you feel mentally prepared?

7. How old were you when you had the mastectomy?
    - Do you think this experience would have been different if you would have been a different age?
8. What happened after the treatment?
    - What changes occurred in your life after the treatment?
    - Did you notice any changes in people's behavior toward you?
    - What are your thoughts about the physical changes in your body after the treatment?
    - Do you feel healthy after the treatment?
9. Many people feel like it is still a taboo to talk about mastectomies. What do you think about that?
    - Did you encounter any reactions when people learned you had undergone mastectomy?
    - Did you talk to people about it openly?

Final Reflection:

How would you describe your journey, and what do you think are the things in our society that others can learn from the lives of strong individuals like you, and how can we impart that lesson to them?
